# Comparison of PIV and Other Immune Inflammation Markers of Oncological and Survival Outcomes in Patients Undergoing Radical Cystectomy

**DOI:** 10.3390/cancers16030651

**Published:** 2024-02-03

**Authors:** Pierluigi Russo, Giuseppe Palermo, Roberto Iacovelli, Mauro Ragonese, Chiara Ciccarese, Giuseppe Maioriello, Fabrizio Fantasia, Francesco Pio Bizzarri, Filippo Marino, Koosha Moosavi, Domenico Nigro, Giovanni Battista Filomena, Filippo Gavi, Francesco Rossi, Francesco Pinto, Marco Racioppi, Nazario Foschi

**Affiliations:** 1 Department of Urology, Fondazione Policlinico Universitario Agostino Gemelli, Largo Francesco Vito 1, 00168 Rome, Italy; pierluigi.russo01@icatt.it (P.R.); mauroragonese@yahoo.it (M.R.); giuseppemaioriello98@gmail.com (G.M.); fabriziof91@hotmail.it (F.F.); francescopiobizzarri1994@gmail.com (F.P.B.); dr.filippomarino@gmail.com (F.M.); koosha96@hotmail.it (K.M.); dno.nigro@gmail.com (D.N.); giovanni.filomena17@gmail.com (G.B.F.); filippogavimd@gmail.com (F.G.); francescorossi1010@gmail.com (F.R.); francesco.pinto@policlinicogemelli.it (F.P.); marco.racioppi@policlinicogemelli.it (M.R.); nazario.foschi@policlinicogemelli.it (N.F.); 2 Department of Oncology, Fondazione Policlinico Universitario Agostino Gemelli, Largo Francesco Vito 1, 00168 Rome, Italy; roberto.iacovelli@policlinicogemelli.it (R.I.); ciccarese.g@gmail.com (C.C.)

**Keywords:** urothelial carcinoma, inflammation, pan-immune inflammation value, radical cystectomy

## Abstract

**Simple Summary:**

Although considerable progress has been made in the management of high-risk and muscle-invasive bladder neoplasm in terms of new therapeutic techniques and new chemo/radiotherapy treatments, it remains a high-mortality tumor, with about 50% of patients developing distant metastasis. The outlook following radical cystectomy is contingent on histological traits like staging and grading of the tumor, metastatic condition, involvement of lymphatic nodes, histological variant, or lymph vascular invasion and vascular infiltration. It would be advisable to develop a prognostic model and preoperative risk stratification for those patients most at risk who might need further treatment after surgery. Although much research has been conducted on the use of blood biomarkers to improve the follow-up for these patients, there is still much confusion about this, and no biomarker is standard in the clinical setting. Our retrospective research aimed to examine the prospective added value of the pan-immune inflammation value (PIV) index and other known predictive factors and compare them with other inflammation indices for the oncological outcomes of patients treated with radical cystectomy (RC).

**Abstract:**

Inflammation is widely acknowledged as a significant characteristic of cancer, playing a substantial function in both the initiation and advancement of cancers. In this research, we planned to compare pan-immune inflammation markers and other well-known markers (systemic immune inflammation index and neutrophil to lymphocyte ratio) to predict prognosis in individuals treated with radical cystectomy for bladder cancer. Methods: In this retrospective analysis, we focused on preoperative PIV, systemic immune inflammation index (SII), and neutrophil–lymphocyte ratio (NLR) in 193 individuals managed with radical cystectomy for bladder cancer between January 2016 and November 2022. Multivariable logistic regression assessments were performed to assess the predictive capabilities of PIV, SII, and NLR for infiltration of lymph nodes (N), aggressive tumor stage (pT3/pT4), and any non-organ limited disease at the time of RC. Multivariable Cox regression analyses were conducted to assess the predictive impact of PIV on Relapse-free survival (RFS), Cancer-specific survival (CSS), and Overall survival (OS). Results: Our individuals were divided into high PIV and low PIV cohorts using the optimal cut-off value (340.96 × 10^9^/L) based on receiver operating characteristic curve analysis for relapse-free survival. In multivariable preoperative logistic regression models, only SII and PIV correlated with the infiltration of lymph nodes, aggressive disease, and any non-organ confined disease. In multivariable Cox regression models considering presurgical clinicopathological variables, a higher PIV was associated with diminished RFS (*p* = 0.017) and OS (*p* = 0.029). In addition, in multivariable Cox regression models for postoperative outcomes, a high PIV correlated with both RFS (*p* = 0.034) and OS (*p* = 0.048). Conclusions: Our study suggests that PIV and SII are two very similar markers that may serve as independent and significant predictors of aggressive disease and worse survival impacts on individuals undergoing radical cystectomy for bladder neoplasm.

## 1. Introduction

Bladder cancer, a frequently encountered urological malignancy, is a disease of complex management that imposes a significant burden on society, with an annual global diagnosis exceeding 430,000 men and women and occupying 10th place among the most common cancers worldwide [1]. Bladder cancer arises from the transitional epithelium, with urothelial bladder cancer being the predominant subtype, representing over 90% of cases [2]. Non-muscle invasive bladder cancer (NMIBC) is highly prevalent, attributed to its indolent natural course (which occurs in approximately 75% of patients) and frequent recurrence. One-quarter of bladder cancer cases, along with the majority of non-urothelial carcinoma subtypes, progress to the muscle-invasive stage (MIBC). The management of muscle-invasive cases often involves systemic chemotherapy and/or immunotherapy, radical interventions such as cystectomy or radiotherapy, or palliative care [3]. Although various innovative treatment modalities have surfaced in recent years, including antibody–drug conjugates, targeted therapy, and immunotherapy utilizing checkpoint inhibition, a significant portion of patients develop disease escalation within a five-year period, attributed to the elevated incidence of micrometastases [4,5,6]. To date, due to this cancer’s devious behavior at the time of diagnosis and its inaccurate preoperative clinical staging, only postoperative pathologic features offer a dependable prognostic assessment. In this context, the knowledge and evaluation of potential biomarkers or predictive models play a crucial role in stratifying risks, planning treatments, closely monitoring patients, and determining oncological outcomes. As observed in various neoplasms, immune response cells (neutrophils, monocytes, and lymphocytes), platelets, and their associate signaling pathways constitute crucial elements within the tumor microenvironment, significantly influencing tumor progression and metastasis [7]. Many of these indicators, such as systemic immune inflammation index (SII), platelet-to-lymphocyte ratio (PLR), lymphocyte-to-monocyte ratio (MLR), and systemic inflammation score, have been reported to forecast the aggressive form of the disease and survival outcomes in various types of neoplasms, including BCa [8,9,10]. Many studies have focused more on these two biomarkers, NLR and SII. Regarding NLR, a meta-analysis of 23 papers has highlighted that high NLR is linked to poorer global survival (OS), cancer-specific survival (CSS), and relapse-free survival (RFS) in patients affected by urothelial cancer [11]. An intensified neutrophil response and concurrent suppression of lymphocytes, reflected in a high NLR, have the potential to foster carcinogenesis and hamper the anti-tumor immune response. 

The systemic immune inflammation index (SII)—evaluated using the following equation: neutrophil count multiplied by platelet count, divided by lymphocyte count—assesses patients’ inflammatory and immune response considering three cell lines that play a vital role in the immune process. High SII is closely tied to adverse outcomes and more hostile pathological traits in several types of cancer, including bladder cancer [12,13,14]. Recently, the pan-immune inflammation value (PIV), which is based on peripheral monocytes, platelets, neutrophils, and lymphocytes, has been employed as an evaluation tool for the preoperative balance of inflammatory factors and immune status, enhancing the ability to make a more accurate prognosis prediction and with a role that has been clarified in the literature. Our objective was to compare PIV with two other biomarkers commonly used today, SII and NLR, to provide essential insights into the potential association between PIV and adverse cancer-related events within a uniformly characterized and precisely characterized patient cohort. Another well-known risk factor associated with an increased likelihood of BCa recurrence and progression is age. Indeed, as demonstrated by Ferro et al. [15], patients older than 70 years and diagnosed with Carcinoma in situ (CIS) had a lower response to BCG therapy and an increased risk of CIS recurrence and progression. Our single-center and retrospective investigation was designed to assess the prospective preoperative role of PIV as an indicator for malignant conditions and adverse results. 

## 2. Materials and Methods

### 2.1. Patients

The records of 314 patients who underwent radical cystectomy and lymphadenectomy at the hospital of the researcher from January 2016 to November 2022 were examined. We only evaluated patients with non-metastatic urothelial bladder cancer and did not consider other histologic variants. The data collection followed the principles outlined in the Declaration of Helsinki and obtained endorsement from the Institutional Ethical Board. Of all patients who underwent RC, we excluded those who could have an altered inflammatory/immune profile independent of disease and surgery. In particular, patients with previous radiotherapy of the pelvis (3), previous surgery (1), neoadjuvant chemotherapy (115), and a history of autoimmune disease (2) were excluded from our analysis. Ultimately, this retrospective study included 193 enrolled patients, all of whom underwent both open and robotic radical cystectomy (RC) along with lymphadenectomy. The selection of lymphadenectomy extent and the method of urinary reconstruction were determined considering the individual’s medical traits and the doctor’s discernment. Before RC, we performed routine presurgical examinations, clinical and physical tests, and imaging studies (computer tomography or magnetic resonance) to rule out metastases. The RC specimens were examined by a specialized uropathologist who staged them according to the 2017 classification (8th edition) of the Tumor Nodes Metastasis (TNM) staging. The histological grading was also reviewed using the 2004/2016 World Health Organization system.

### 2.2. Data Gathering

Patient features recorded prior to surgery were age, sex, smoking condition, diabetes, body composition index or BMI, clinical staging (cT), operative technique, urinary reconstruction, tumor grading (pT), nodal status (pN), lymph vascular infiltration (LVI), and adjuvant therapy. Laboratory examinations included neutrophil count (10^9^/L), platelet count (10^9^/L), monocyte count (10^9^/L), and lymphocyte count (10^9^/L). PIV, SII, and NLR were the results of “neutrophil count × platelet count × monocyte count/lymphocyte count”, “neutrophil count × platelet count/lymphocyte count”, and “neutrophil count/lymphocyte count”, respectively. The PIV value was derived via constructing a diagnostic performance curve (ROC), utilizing relapse as the outcome to optimize the Youden index. Subsequently, individuals were categorized into low PIV and high PIV groups, determined by the optimal cut-off PIV value (<340.96 and >340.96, respectively).

### 2.3. Outcomes

Association between inflammatory biomarkers and unfavorable pathological characteristics, including lymph node infiltration, advanced tumor grading (pTstage), and locoregionally extended state (defined as any progressed pT stage and lymphatic infiltration) at RC pathology were established as the primary outcomes of our study. As secondary outcomes, we analyzed survival outcomes as relapse-free survival (RFS), cancer-specific survival (CSS), and overall survival (OS). We defined RFS as the time from radical cystectomy (RC) to the first occurrence of local relapse or systemic spread. CSS and OS were established as the duration from surgery to death from neoplasm and general mortality, respectively. 

### 2.4. Statistical Methodology 

Statistical analysis was performed using STATA/SE version 18 (StataCorp, College Station, TX, USA). With the ROC curve and the Youden index, we obtained the best cut-offs for each of the biomarkers analyzed. Descriptive statistics were reported as medians and interquartile ranges (IQR) for continuous variables, while frequencies and percentages were reported as categorical variables. The assessment of patient characteristics involved employing the Mann–Whitney U test for continuous data and utilizing the chi-square test and Fisher’s exact tests for categorical data. Statistical significance was defined as a *p*-value below 0.05. Correlation between preoperative features (lymph node infiltration, advanced tumor grading (pT3/pT4), and locoregionally extended state) and PIV, SII, and NLR were examined through univariable and multivariable logistic regression models. To gauge the predictive efficacy of each model, we determined the Area under the Curve (AUC) from the ROC analysis. At the same time, we applied the Hosmer–Lemeshow test to appraise the model’s effectiveness. Relapse-free survival rate (RFS), Cancer-specific survival rate (CSS), and Overall survival rate (OS) were established using the Kaplan–Meier, and the relationship between RFS, CSS, and OS was analyzed using log-rank tests. Univariable and multivariable Cox proportional hazard regression models were conducted to determine the correlation between PIV and RFS, CSS, and OS. Harrel’s C-index or concordance index was used to evaluate the discriminatory power of models. With the likelihood ratio test, we compared the goodness of fit of two statistical models, where one model was a restricted version of the other (model without PIV).

## 3. Results

### 3.1. Patient Features

**Figure 1:**
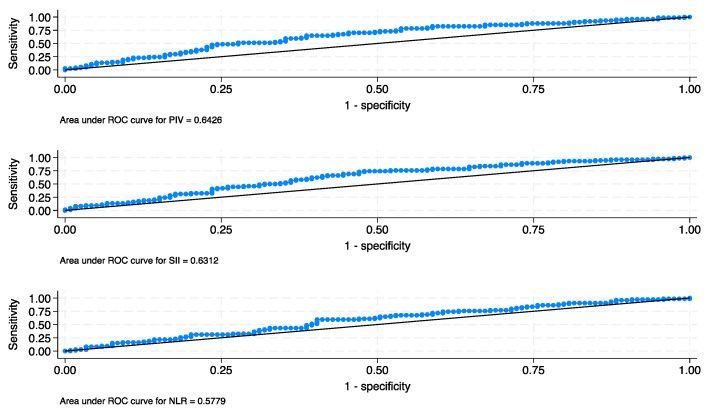
ROC curves of PIV, SII, NLR for RFS.

The demographic features of the patients stratified into the two PIV groups are outlined in Table 1. The median estimates of NLR, SII, and PIV (×10^9^) were 3.0 (quartile 2.1–4.3), 712.5 (quartile 460.5–1215.8), and 311.4 (quartile 185.5–644.5), respectively. The optimal cut-off values of NLR, SII, and PIV (×10^9^) were 3.14, 640.27, and 340.96, respectively. The corresponding AUC for the three markers was 0.57, 0.63, and 0.65, respectively (Figure 1). An elevated presurgical PIV was more frequently detected in individuals with conditions characterized by more advanced clinical (*p* < 0.001) and histological stages (*p* = 0.001), lymph node involvement (*p* = 0.004), lymph vascular invasion (*p* < 0.001), locoregionally extended state (*p* < 0.001), and disease progression (*p* = 0.004). 

### 3.2. Oncological Results 

Table 2, Table 3 and Table 4 show the results of logistic regression analyses (both univariable and multivariable) concerning the prediction of oncological outcomes of three biomarkers. Elevated PIV was linked to an increased likelihood of infiltration of lymph nodes (odds ratio (OR) 1.11, 95% confidence interval (95% CI): 0.34–1.89; *p* = 0.005), advanced Tumor grading (pT3/pT4) (OR 1.17, 95% CI: 0.58–1.76; *p* < 0.001), and locoregionally extended state (OR 1.32, 95% CI: 0.72–1.92; *p* < 0.001). We obtained different results regarding the NLR. High NLR correlated with aggressive pT stage (OR 0.63, 95% CI: 0.06–1.21; *p* = 0.02). High SII correlated with an increased likelihood of nodal invasion (OR 1.35, 95% CI: 0.47–2.23; *p* = 0.003), advanced Tumor grading (OR 0.81, 95% CI: 0.22–1.40; *p* = 0.007) and locoregionally extended state (OR 1.11, 95% CI: 0.52–1.71; *p* < 0.001). 

In multivariable logistic regression models, adjustments were made for presurgical features (age, sex, smoke, and disease staging), and an elevated PIV maintained an independent association with advanced tumor grading (OR 2.87, 95% CI: 1.36–6.04; *p* = 0.005) and locoregionally extended state (OR 3.30, 95% CI: 1.60–6.77; *p* = 0.001). SII also showed a statistically independent association with lymph node infiltration (OR 2.91, 95% CI: 1.09–7.72; *p* = 0.03), complex tumor grading (OR 2.09, 95% CI: 1.00–4.40; *p* = 0.05), and locoregionally extended state (OR 2.96, 95% CI: 1.44–6.08; *p* = 0.003). Table 3 shows no significant correlation between NLR and the three variables in the multivariable logistic regression analysis.

### 3.3. Long-Term Outcomes

**Figure 2:**
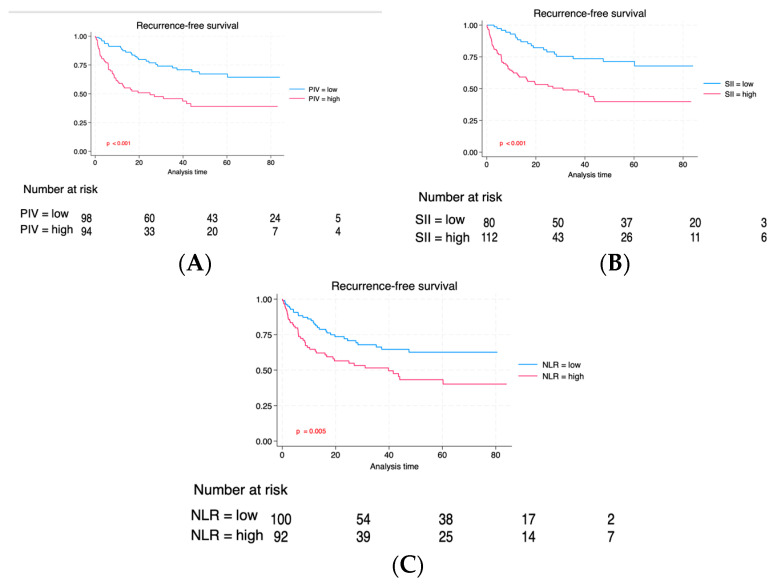
Kaplan–Meier of RFS is based on baseline PIV (**A**), SII (**B**), and NLR (**C**).

**Figure 3:**
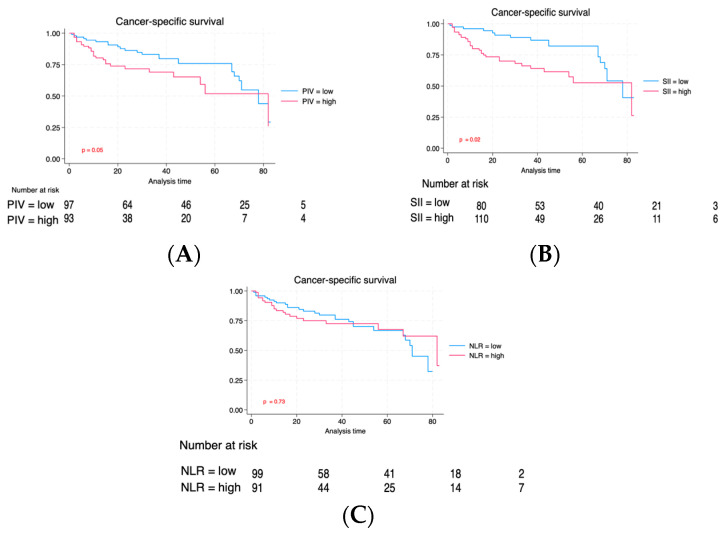
Kaplan–Meier of CSS is based on baseline PIV (**A**), SII (**B**), and NLR (**C**).

**Figure 4:**
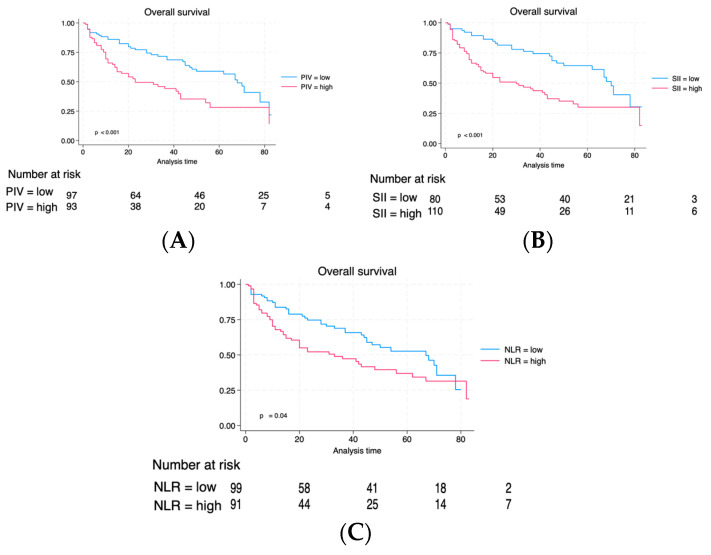
Kaplan–Meier of OS is based on baseline PIV (**A**), SII (**B**), and NLR (**C**).

Individuals with elevated PIV experienced a reduced follow-up duration (36 vs. 23 months, *p* < 0.001). Likewise, individuals exhibiting a high SII also exhibited a reduced median follow-up duration (24 vs. 37 months, *p* = 0.002). Although patients with high NLR exhibited a shorter median follow-up, the difference did not reach statistical significance (27 vs. 32 months, *p* = 0.14). Through the follow-up duration, recurrences occurred in 74 (38.3%) individuals, and 96 (49.7%) patients succumbed, with 52 (26.9%) of these fatalities linked to neoplasm. Figure 2 compares RFS between PIV, SII, and NLR groups. The Kaplan–Meier estimate curves revealed significantly poorer survival outcomes across all three biomarkers regarding relapse-free interval (RFS). Figure 3 illustrates a correlation regarding Cancer-specific Survival (CSS) for PIV, SII, and NLR. Individuals exhibiting elevated PIV and SII levels experienced a notably diminished Cancer-specific survival rate (CSS) compared to those with lower PIV and SII levels (*p* = 0.05 and *p* = 0.02), respectively. There was no significant difference for CSS regarding the NLR biomarker between high NLR and low NLR. Lastly, we evaluated the overall survival among the three groups (Figure 4). As can be seen in Figure 4, individuals with an elevated PIV, SII, and NLR had lower overall survival than those with lower values (*p* < 0.001, *p* < 0.001, and *p* = 0.04), respectively.

We employed a Cox regression model to explore the relationship between the PIV value and time to the event of interest. Initially, we assessed the PIV’s effect considering preoperative variables, including smoke, gender, and clinical T stage. In this kind of analysis, an elevated PIV was significantly linked with RFS (hazard ratio (HR): 1.89, 95% CI: 1.12–3.19; *p* = 0.017) and global survival (OS) (HR: 1.64, 95% CI: 1.05–2.56; *p* = 0.029) (Table 5).

Subsequently, we examined how this association changed when introducing postoperative variables, such as pT stage, lymph vascular invasion, lymph node infiltration, and adjuvant chemotherapy. Also, in this case, high PIV was strongly associated with RFS (HR 1.74, 95% CI: 1.04–2.92; *p* = 0.034) and OS (HR 1.58, 95% CI: 1.00–2.49; *p* = 0.048) (Table 6). Despite the addition of a preoperative PIV to the reference model not yielding a significant discrimination potential of the model for CSS (*p* = 0.67), it did, however, result in a considerable improvement of both RFS (by 1%, *p* = 0.03) and OS (by 1%, *p* =0.04).

## 4. Discussion

We analyzed the prognostic implications of preoperative PIV and compared its effects with other biomarkers in patients undergoing RC for BCa. The assessment and recognition of systemic variables that could indicate a poorer prognosis in patients with various diseases have been the subject of growing research [16]. Our study represents the first analysis in existing literature encompassing an evaluation of PIV with other distinct systemic indices associated with an unfavorable prognosis among patients with BCa. In a previous article, it was reported that high PIV in individuals with non-metastatic MIBC was linked to worse global survival and cancer-specific survival [17]. Conforming to the findings of the only article in the literature [17], an elevated PIV was related to increased aggressiveness of urothelial bladder cancer (worst clinical T stage, lymph node infiltration, lymph vascular involvement, progressive disease) and continued to be independently linked to pT3/T4 disease, locoregional advanced disease, and lymph node metastasis. Also, our analysis revealed that high PIV (>340.96) demonstrated an independent correlation with survival results (both RFS and OS) in a model that accounted for both pre- and post-surgical factors. The PIV and SII exhibited better predictive power than NLR. As far as our understanding goes, this study is the initial observation of an association within a logistic model that integrates postsurgical features. Furthermore, the study reveals the intricate interplay between inflammation and cancer, which is a pivotal aspect underpinning the complex dynamics of tumorigenesis and disease progression. Inflammatory responses mirror the systemic immune status, unveiling the intricacies of compromised immune surveillance induced by cancer cells [18]. The biomarkers analyzed in this study comprise cells that are key players in the body’s immune and inflammatory processes. Neutrophils and platelets have been demonstrated to facilitate the advancement of neoplasm [19]. Neutrophils promote cellular cohesion, acting as a bridge as they bind circulating tumor cells (CTCs) to the endothelium of the organ in which they are located (a contact-dependent mechanism) [20]. In addition, neutrophils can release soluble components that stimulate endothelial and parenchymal cells, thereby augmenting the attachment of CTCs in distant organs [21,22,23]. Platelets, through the release of dense adenine nucleotide granules in the tumor stroma, assist the migration of tumor cells across the endothelium and, in addition, support tumor cell survival in the bloodstream and induce a pro-metastatic phenotype [24]. Monocytes play a crucial role in tumor development and metastasis. Tumor-associated macrophages derived from peripheral blood mononuclear cells can suppress the acquired immune response [25]. Conversely, lymphocytes can exert an anti-tumoral function by promoting cellular cytotoxicity, impeding tumor cell replication, and bolstering the organism’s immune reaction against tumors [26,27]. It has already been shown in the literature that NLR is an unfavorable prognostic indicator in patients diagnosed with urothelial bladder cancer, and a high NLR negatively correlates with DFS [28]. Another meta-analysis by Tang et al., which included 5425 patients across 17 studies, showed that NLR had negative predictive value for OS, CSS, RFS, and PFS [29]. Our findings revealed a statistically significant negative correlation between NLR and OS but not for CSS and DFS. Moreover, NLR had lower predictive power among the three biomarkers used than SII and PIV for oncological and survival outcomes. Another factor examined was the systemic immune inflammation index, which has become increasingly popular recently. The SII has been employed as a prognostic marker in urological cancers and other malignancies. Jian-Hui C. et al. showed a relationship between SII and colorectal cancer [30], Wang et al. detected a correlation between elevated SII and lung neoplasm [31], and Zhong et al. discovered a strong connection between an elevated SII and a poorer prognosis in solid tumors [32]. In particular, in bladder cancer, an elevated SII has been linked to worse survival and oncological outcomes [33,34]. In addition, the superiority of SII over other biomarkers, such as NLR and PLR, has already been demonstrated in the prognosis of oncological disease [35,36,37]. Our findings are consistent with the existing literature, illustrating that an elevated SII adversely impacts both RFS and OS. Finally, the last biomarker evaluated and compared with the others was the pan-immune inflammation value, a newly defined inflammation-related index. In 2020, the investigation of PIV commenced in patients with metastatic colorectal cancer, revealing its substantial prognostic value for patient survival. Furthermore, PIV’s predictive efficiency was significantly superior to previously recognized inflammation-related markers [38]. A meta-analysis conducted by Qi et al., involving eight articles and 2953 patients with breast cancer, showed that elevated PIV in patients correlated with shorter OS and PFS [39]. In the context of bladder cancer specifically, Kayar et al. discovered that an elevated PIV (>406.29) was associated with diminished OS and DFS [17]. Our analysis revealed that patients with higher PIV (>340.96) had a worse RFS and OS in both Cox regression models incorporating pre- and post-operative characteristics. In addition, the biomarker PIV correlated very similarly with SII with oncological and survival outcomes. These data hold the capability to improve predictive models, resulting in a more precise prognosis for patients undergoing RC. Nonetheless, establishing a clinical utility for PIV as a biomarker goes beyond merely associating high PIV with aggressive disease or compromised survival results in multivariable models [40]. High PIV value may result from other immune/inflammatory pathologies such as chronic inflammation, autoimmune disease, or infectious disease. Nevertheless, further examination of PIV is justified, given its immediate and economical accessibility. The key significance of this analysis resides in its potential benefit for practitioners as a supplementary indicator to assess the prognosis of BCa. This can improve the precision of risk assessment and contribute to more precise treatment-planning decisions, including the evaluation of adjuvant therapy, neoadjuvant therapy, or bladder-sparing therapies. Despite the fact that chronic inflammation is a risk factor for a worse prognosis for a cancer patient and despite sharing similar biological processes with cardiovascular disease (CVD), it has been shown by Barone et al. [41] that the presence of CVD is an independent protective factor for BCa, at least for low- and intermediate-risk BCa but not for high-risk BCa.

While our study provides valuable insights for the first time, it is essential to recognize its limitations. The primary challenges revolve around the limitations associated with retrospective data collection, small sample size, and monocentric cohort study. This analysis is strengthened by the deliberate exclusion of potential confounding factors related to PIV, the consistent use of the same laboratory for PIV evaluations, and histological examinations conducted by a consistent uropathologist. Although akin to approaches applied in previous studies, the optimal PIV cut-off value for BC patients still needs to be determined. It will be crucial to validate our findings externally using independent cohorts to confirm the relevance and applicability of our results.

## 5. Conclusions

In conclusion, our study suggests that both PIV and SII can serve as independent predictors of aggressive disease and prognosis in bladder cancer patients following radical cystectomy. Looking ahead, the incorporation of SII and PIV into clinical practice holds promise for enhancing diagnostic and prognostic strategies. The ability to identify patients with a more aggressive disease profile early on may guide tailored therapeutic interventions and improve overall clinical results. Further investigation and validation studies are justified to strengthen the applicability of these biomarkers and to explore their potential in personalized medicine and treatment optimization.

## Figures and Tables

**Table 1:** Analyzing the differences in patients’ demographics between those with high PIV and low PIV.

Characteristic	Total	Low PIV	High PIV	*p*
	N = 193	N = 99 (51.3)	N = 94 (48.7)	
Age, median (IQR)	78 (73–83)	78 (73–83)	79 (73–83)	0.38
Sex, *n* (%)				0.07
Male	154 (79.7)	84 (84.8)	70 (74.7)	
Female	39 (20.2)	15 (15.1)	24 (25.5)	
Smoke, *n* (%)	149 (77.2)	83 (83.8)	66 (70.2)	0.02
Diabetes, *n* (%)	31 (16.0)	16 (16.1)	15 (15.9)	0.96
Clinical T stage, *n* (%)				<0.001
cTa	67 (34.7)	41 (41.4)	26 (27.6)	
cTis	24 (12.4)	18 (12.3)	6 (11.7)	
cT1	49 (25.3)	23 (25.1)	26 (23.9)	
cT2	29 (15.0)	14 (14.9)	15 (14.1)	
cT3	18 (9.3)	3 (9.2)	15 (8.8)	
cT4	6 (3.1)	0 (3.1)	6 (2.9)	
BMI, median (IQR)	26 (24–29)	26 (24–29)	26 (24–28)	0.39
Surgical approach, *n* (%)				0.27
Open	186 (96.3)	94 (94.9)	92 (97.8)	
Robot-assisted	7 (3.6)	5 (5.0)	2 (2.1)	
Urinary diversion, *n* (%)				0.003
Ureterocutaneostomy	21 (10.8)	6 (6.0)	15 (15.9)	
Ileal conduit	148 (76.6)	74 (78.2)	74 (78.7)	
Orthotopic neobladder	24 (12.4)	19 (19.1)	5 (5.3)	
Pathological T stage, *n* (%)				0.001
pT0	14 (7.2)	10 (10.1)	4 (4.2)	
pTa	10 (5.1)	(5.0)	5 (5.3)	
pTis	23 (11.9)	17 (17.1)	6 (6.3)	
pT1	26 (13.4)	19 (19.1)	7 (7.4)	
pT2a	30 (15.5)	16 (16.1)	14 (14.8)	
pT2b	5 (2.5)	3 (3.0)	2 (2.1)	
pT3a	49 (25.3)	22 (22.2)	27 (28.7)	
pT3b	8 (4.1)	4 (3.0)	5 (5.3)	
pT4a	21 (10.8)	4 (4.0)	17 (18.0)	
pT4b	7 (3.6)	0 (0.0)	7 (7.4)	
Lymph node, *n* (%)	37 (19.1)	11 (11.1)	26 (27.6)	0.004
LVI, *n* (%)	128 (66.3)	54 (54.5)	74 (78.7)	<0.001
Locally advanced disease, *n* (%)	102 (52.8)	37 (37.3)	65 (69.1)	<0.001
Adjuvant chemotherapy, *n* (%)	40 (20.7)	15 (15.1)	25 (26.6)	0.05
Progressive disease, *n* (%)	61 (31.6)	22 (22.2)	39 (41.4)	0.004
Cancer-related deaths, *n* (%)	96 (49.7)	44 (44.4)	52 (55.3)	0.131
Any-cause deaths, *n* (%)	52 (26.9)	26 (26.2)	26 (27.6)	0.827

PIV = Pan-immune inflammation index; BMI = Body mass index; IQR = Interquartile range; LVI = lymph vascular infiltration.

**Table 2:** Analyzing the relationship between PIV and oncological outcomes using multivariable logistic regression.

		Lymph Node			Advanced Tumor Grading			Locoregionally Extended State	
Characteristic	OR	95% CI	*p*-value	OR	95% CI	*p*-value	OR	95% CI	*p*-value
PIV (Reference: low)									
High	1.84	0.76, 4.47	0.17	2.87	1.36, 6.04	0.005	3.30	1.60, 6.77	0.001
Age	1.14	0.33, 3.88	0.82	0.75	0.28, 1.99	0.576	1.00	0.39, 2.57	0.98
Smoke (Reference: no)									
Smoke	0.59	0.23, 1.50	0.27	0.93	0.37, 2.33	0.890	1.48	0.59, 3.70	0.39
Sex (Reference: male)									
Female	0.56	0.19, 1.60	0.28	0.31	0.10, 0.90	0.032	0.53	0.20, 1.42	0.21
Clinical tumor stage									
(Reference: cTa/cTis/cT1)									
cT2	4.92	1.84, 13.13	0.001	45.10	9.46, 215.03	<0.001	29.15	6.32, 134.36	<0.001
cT3/cT4	11.16	3.86, 32.27	<0.001	23.49	4.91, 112.20	<0.001			
Goodness-of-fit test	Hosmer–Lemeshow test		0.20			0.68			0.91
AUC									
Model with PIV		AUC: 0.78			AUC: 0.80			AUC: 0.81	
Model without PIV		AUC: 0.76 (+2%)			AUC: 0.77 (+3%)			AUC: 0.76 (+5%)	
*(p =* difference model)			0.27			0.12			0.04

PIV = Pan-immune inflammation value; OR = Odds Ratio; CI = Confidence Inter al; AUC = Area under the curve.

**Table 3:** Analyzing the relationship between NLR and oncological outcomes using multivariable logistic regression.

		Lymph Node			Advanced Tumor Grading			Locoregionally Extended State	
Characteristic	OR	95% CI	*p*-value	OR	95% CI	*p*-value	OR	95% CI	*p*-value
NLR (Reference: low)									
High	1.47	0.65, 3.36	0.35	1.98	0.97, 4.05	0.06	1.60	0.80, 3.18	0.17
Age	1.13	0.33, 3.82	0.84	0.76	0.29, 1.99	0.588	1.01	0.40, 2.51	0.97
Smoke (Reference: no)									
Smoke	0.54	0.21, 1.35	0.19	0.79	0.32, 1.94	0.620	1.22	0.51, 2.93	0.65
Sex (Reference: male)									
Female	0.58	0.20, 1.66	0.31	0.31	0.11, 0.92	0.035	0.57	0.21, 1.48	0.25
Clinical tumor stage									
(Reference: cTa/cTis/cT1)									
cT2	4.99	1.88, 13.25	0.001	44.10	9.29, 209.21	<0.001	27.01	5.97, 122.05	<0.001
cT3/cT4	13.19	4.72, 36.85	<0.001	32.22	6.73, 154.29	<0.001			
Goodness-of-fit test	Hosmer–Lemeshow test		0.97			0.25			0.91
AUC									
Model with NLR		AUC: 0.77			AUC: 0.78			AUC: 0.77	
Model without NLR		AUC: 0.76 (+1%)			AUC: 0.77 (+1%)			AUC: 0.76 (+1%)	
*(p =* difference model)			0.51			0.55			0.55

NLR = Neutrophil to lymphocyte ratio; OR = Odds ratio; CI = Confidence interval; AUC = Area under the curve.

**Table 4:** Analyzing the relationship between SII and oncological outcomes using multivariable logistic regression.

		Lymph Node			Advanced Tumor Grading			Locoregionally Extended State	
Characteristic	OR	95% CI	*p*-value	OR	95% CI	*p*-value	OR	95% CI	*p*-value
SII (Reference: low)									
High	2.91	1.09, 7.72	0.03	2.09	1.00, 4.40	0.05	2.96	1.44, 6.08	0.003
Age	1.10	0.33, 3.72	0.86	0.79	0.30, 2.07	0.644	1.06	0.42, 2.70	0.89
Smoke (Reference: no)									
Smoke	0.56	0.22, 1.41	0.22	0.82	0.33, 2.02	0.680	1.30	0.53, 3.19	0.56
Sex (Reference: male)									
Female	0.52	0.18, 1.51	0.23	0.31	0.11, 0.91	0.034	0.51	0.19, 1.36	0.18
Clinical tumor stage									
(Reference: cTa/cTis/cT1)									
cT2	5.35	1.97, 14.56	0.001	45.06	9.47, 214.31	<0.001	31.41	6.73, 146.46	<0.001
cT3/cT4	10.58	3.70, 30.19	<0.001	27.08	5.66, 129.51	<0.001			
Goodness-of-fit test	Hosmer–Lemeshow test		0.89			0.21			0.82
AUC									
Model with SII		AUC: 0.79			AUC: 0.80			AUC: 0.81	
Model without SII		AUC: 0.76 (+3%)			AUC: 0.77 (+3%)			AUC: 0.76 (+5%)	
*(p =* difference model)			0.17			0.16			0.03

SII = Systemic immune inflammation index; OR = Odds ratio; CI = Confidence interval; AUC: Area under the curve.

**Table 5:** Multivariable Cox regression analysis to predict RFS, CSS, and OS before surgery.

		Relapse-Free Survival			Cancer-Specific Survival			Overall Survival	
	HR	95% CI	*p*-value	HR	95% CI	*p*-value	HR	95% CI	*p*-value
PIV (Reference: low)									
High	1.89	1.12, 3.19	0.017	1.07	0.57, 2.02	0.819	1.64	1.05, 2.56	0.029
Age	0.77	0.40, 1.46	0.427	1.27	0.53, 3.04	0.579	1.11	0.61, 2.02	0.717
Smoking status (Reference: no)									
Smoke	0.91	0.53, 1.55	0.744	1.30	0.62, 2.73	0.478	1.25	0.74, 2.10	0.401
Sex (Reference: male)									
Female	0.86	0.49, 1.52	0.624	1.47	0.78, 2.80	0.230	1.06	0.64, 1.75	0.811
Clinical tumor condition									
(Reference: cTa/cTis/cT1)									
cT2	1.58	0.82, 3.05	0.168	1.03	0.42, 2.53	0.938	1.25	0.69, 2.27	0.450
cT3/cT4	7.14	3.90, 13.06	<0.001	8.77	4.13, 18.61	<0.001	4.02	2.24, 7.19	<0.001
Harrel’s index									
Model accuracy			0.72			0.72			0.66
Model accuracy without PIV			0.68 (+4%)			0.71 (+1%)			0.63 (+3%)
(*p* = difference model)			0.01			0.81			0.03

PIV = Pan-immune inflammation value; HR = Hazard ratio; CI = Confidence interval.

**Table 6:** Multivariable Cox regression analysis following surgery to predict RFS, CSS, and OS.

		Relapse-Free Survival			Cancer-Specific Survival			Overall Survival	
Characteristic	HR	95% CI	*p*-value	HR	95% CI	*p*-value	HR	95% CI	*p*-value
PIV (Reference: low)									
High	1.74	1.04, 2.92	0.034	1.30	0.69, 2.43	0.412	1.58	1.00, 2.49	0.048
Age	0.99	0.52, 1.88	0.994	1.46	0.61, 3.49	0.387	1.21	0.67, 2.21	0.517
Smoking status (Reference: no)									
Smoke	1.26	0.72, 2.19	0.403	2.13	1.02, 4.44	0.043	1.73	1.02, 2.92	0.039
Sex (Reference: male)									
Female	1.42	0.77, 2.61	0.258	1.78	0.92, 3.45	0.084	1.30	0.77, 2.18	0.314
Tumor grading									
(Reference: pT0/pTa/pTispT1)									
pT2	1.67	0.65, 4.28	0.283	0.98	0.31, 3.11	0.981	1.54	0.70, 3.40	0.276
pT3/pT4	2.84	1.14, 7.09	0.025	2.88	1.17, 7.11	0.021	3.23	1.59, 6.53	0.001
Lymphovascular invasion	1.03	0.41, 2.54	0.94	0.97	0.38, 2.44	0.957	0.90	0.44, 1.81	0.774
Lymph node invasion	3.85	2.12, 6.99	<0.001	4.26	1.89, 9.61	<0.001	3.12	1.74, 5.60	<0.001
Adjuvant chemotherapy	1.31	0.73, 2.34	0.349	0.44	0.19, 1.00	0.05	0.61	0.34, 1.08	0.092
C-index									
Model with PIV			0.78			0.76			0.73
Model without PIV			0.77 (+1%)			0.76			0.72 (1%)
*(p =* difference model)			0.03			0.67			0.04

PIV = Pan-immune inflammation value; HR = Hazard ratio; CI = Confidence Interval.

## Data Availability

All data used in this analysis were sourced from an anonymized database. The code for the analyses can be provided upon request.
